# Primary Biliary Cirrhosis Overlapping with Autoimmune Hepatitis in an HIV-Infected Patient on Antiretroviral Therapy

**DOI:** 10.5455/jihp.20130624104921

**Published:** 2013-06-30

**Authors:** Margaret Caplan, Apurva Trivedi, Mary McLaughlin, Annick Hebou, David E. Kleiner, Theo Heller, Caryn G. Morse

**Affiliations:** 1 Clinical Research Directorate/CMRP, SAIC-Frederick, Frederick National Laboratory for Cancer Research, Frederick, MD, USA; 2 Liver Diseases Branch, National Institute of Diabetes, Digestive and Kidney Diseases, NIH, Bethesda, MD, USA; 3 Laboratory of Immunoregulation, National Institute of Allergy and Infectious Diseases, NIH, Bethesda, MD, USA; 4 Carl Vogel Center, Washington, DC; USA; 5 Laboratory of Pathology, National Cancer Institute, NIH, Bethesda, MD, USA; 6 Critical Care Medicine Department, NIH Clinical Center, Bethesda, MD, USA

**Keywords:** HIV, overlap syndrome, autoimmune hepatitis, immune reconstitution, liver biopsy

## Abstract

Liver disease in HIV-infected patients is complex and multifactorial. Drug toxicity and infections are common causes of elevations in liver-associated enzymes. Immune reconstitution and unmasking of autoimmune disease may also play a role, particularly in the era of effective combination antiretroviral therapy. In this case report, we describe the first reported biopsy-confirmed case of autoimmune hepatitis and primary biliary cirrhosis overlap syndrome presenting in an HIV-infected patient following initiation of antiretroviral therapy.

## INTRODUCTION

The term “overlap syndrome” describes the rare interface between autoimmune hepatitis (AIH) and primary biliary cirrhosis (PBC) or primary sclerosing cholangitis (PSC). Diagnostic criteria and treatment for this spectrum of autoimmune liver disease remain controversial [1]. Co-existence of Human Immunodeficiency Virus (HIV) infection further complicates diagnosis and treatment in patients with abnormal liver associated enzymes (LAEs). We present the first reported case of a patient with HIV infection and overlapping AIH and PBC in the setting of antiretroviral therapy and immune reconstitution.

## CASE

**Figure 1:**
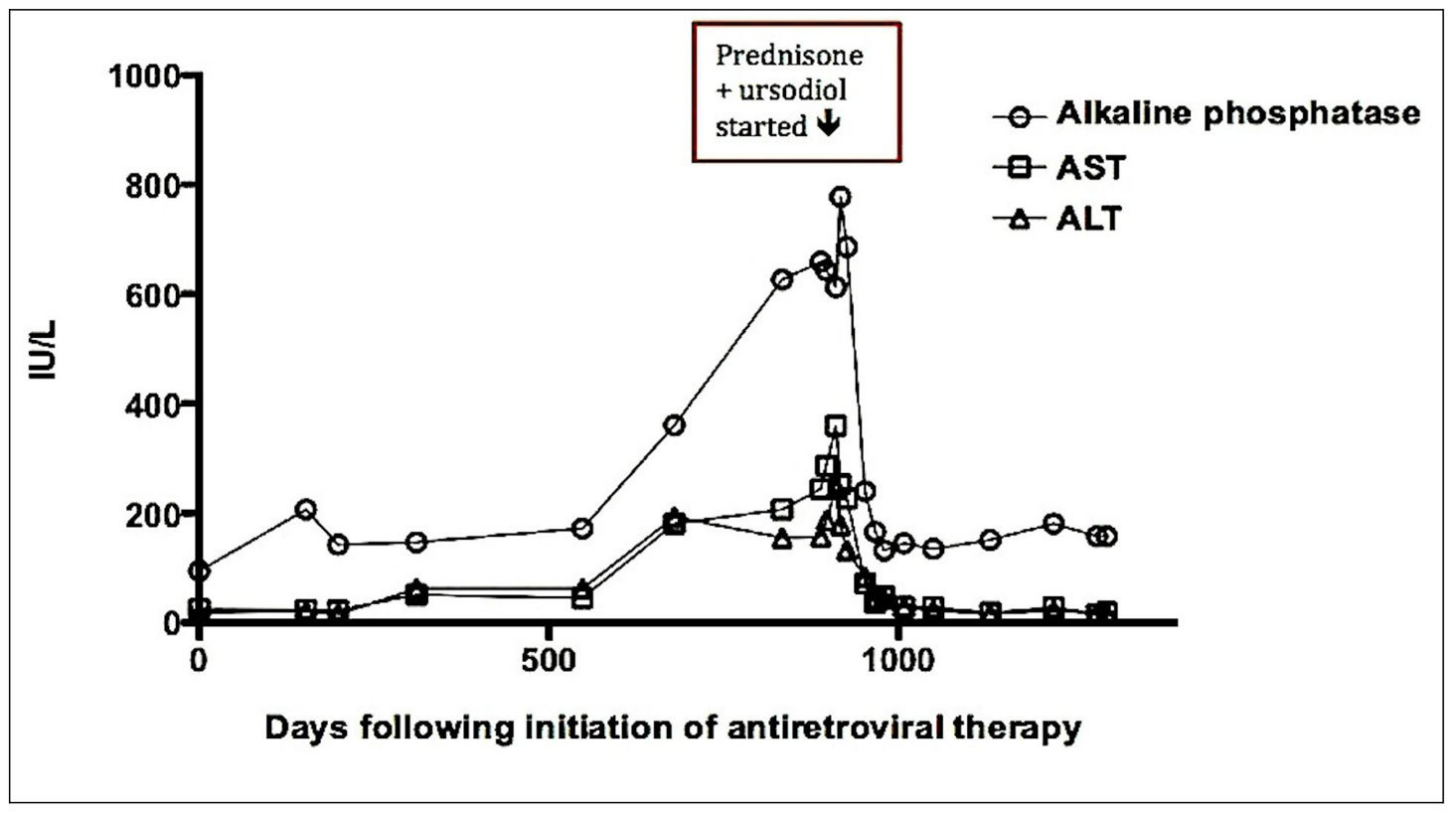
Liver associated enzymes rapidly improve following initiation of anti-inflammatory therapy in a HIV-infected patient with autoimmune hepatitis and primary biliary cirrhosis overlap syndrome.

A 46 year-old African-American woman with HIV infection on antiretroviral therapy (ART) presented to the National Institutes of Health (NIH) from a community health center in Washington, DC, for further evaluation of elevated LAEs. Her baseline CD4 cell count was 420 cells/mm3 and her LAEs were normal prior to starting ART (Figure 1). Five months after initiation of ART with the fixed-dose combination pill of efavirenz/tenofovir/emtricitabine, routine laboratory evaluation revealed a mild increase in her serum alkaline phosphatase (AP) to 207 U/L (normal < 116 U/L). Her AP briefly normalized before increasing again, followed by progressive increases in aspartate aminotransferase (AST) and alanine aminotransferase (ALT) over the next year. Her CD4 cell count increased to 1,100 cells/mm3, with suppression of her HIV viral load from 62,000 copies/mL (log 4.8) to <50 copies/mL within the first 3 months of ART and remained stable thereafter.

On evaluation at the NIH, she complained of mild right upper quadrant pain and denied any other symptom. She reported a family history of scleroderma. She denied known drug allergies, over-the-counter medication use, herbal or dietary supplementation, alcohol abuse or illicit drug use. Screening laboratories showed elevation in her LAEs with AST 244 U/L, ALT 287 U/L, AP of 659 U/L, and gamma glutamyl transferase (GGT) of 607 U/L (Figure 1). Serum total bilirubin peaked at 1.7mg/dL (direct bilirubin 1.3mg/dL). Viral hepatitis serologies, hepatitis B DNA PCR and hepatitis C RNA PCR were negative. Serum iron and ceruloplasmin and alpha-1 antitrypsin levels were normal.

Autoantibody testing was remarkable for a positive ANA, greater than 12.0EU (negative < 1EU, positive > or = 1EU, strongly positive > or = 3EU) and a positive anti-mitochondrial antibody, greater than 0.6U, (negative <0.1U). Anti-smooth muscle antibody and anti-liver-kidney microsomal antibody were negative. Serum copper was elevated slightly above the upper limit of normal (173 mcg/dL, normal <145 mcg/dL). Subsequent laboratory testing revealed hypergammaglobulinemia (serum IgG 5,250 mg/dL, normal <1730 mg/dL), positive anti-centromere (>8.0 U, negative <0.1U) and anti-double stranded DNA (799 IU/mL, normal <30 IU/mL) antibodies. SCL-70 antibodies were negative. Abdominal ultrasound and triple-phase CT scan of the liver were normal.

**Figure 2:**
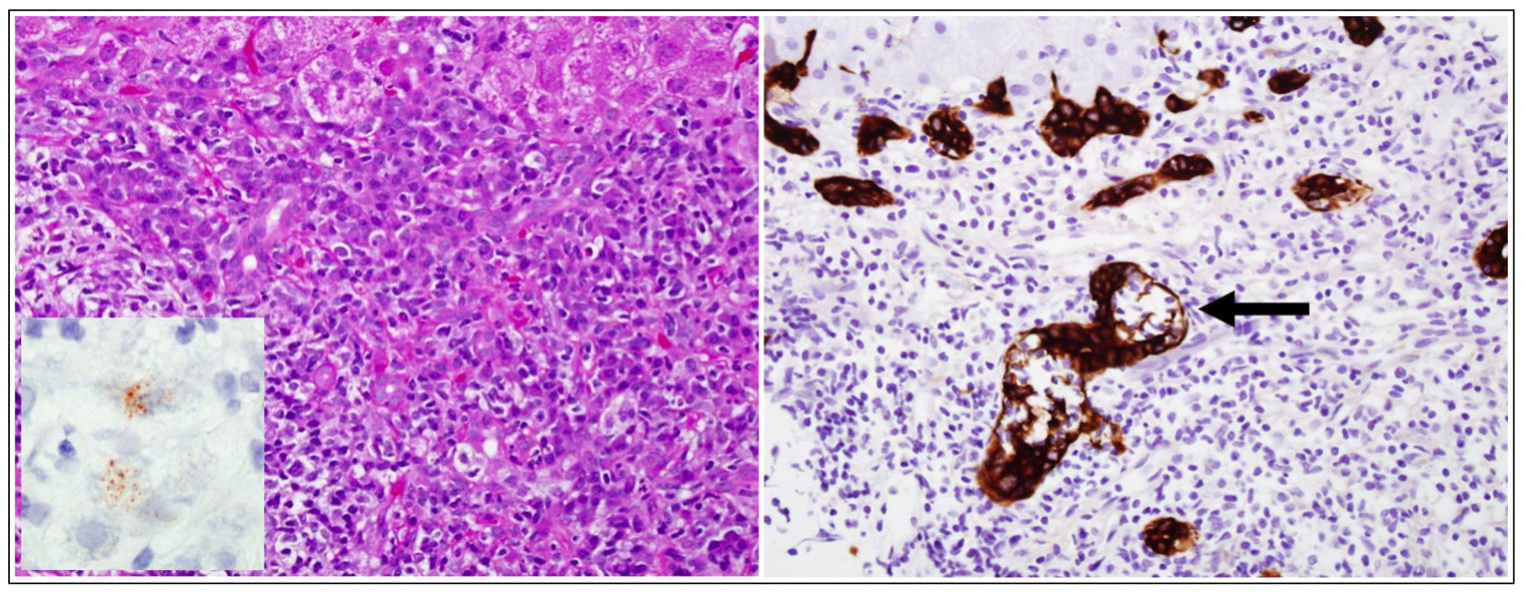
Left: Portal area showing marked chronic inflammation with numerous plasma cells and ductular reaction. Inset shows hepatocytes with a positive staining reaction for copper, indicative of chronic cholestasis. (H&E,x 200; inset: Copper stain, x 600). Right: Cytokeratin 7 stain showing infiltration of a bile duct by lymphocytes (arrow). Ductular reaction is present at the edges of the portal area (CK7 antibody, x400).

Percutaneous liver biopsy showed chronic cholestatic liver disease with marked inflammatory activity, bile duct injury, and periportal and perivenular fibrosis consistent with primary biliary cirrhosis (Figure 2). Immunohistochemistry staining for cytokeratin 7 was performed using anti-cytokeratin 7 antibody from Dako (clone OV-TL-12/30 at 1:400 dilution) and the Ventana Ultra View detection system. Based on this histology and supporting laboratory findings of hypergammaglobulinemia, positive anti-nuclear and anti-mitochondrial antibodies, the patient was diagnosed with primary biliary cirrhosis and autoimmune hepatitis overlap syndrome.

Treatment was initiated with oral prednisone (30mg daily for 7 days, then 20mg daily) and ursodiol (13–15 mg/kg daily). She continued efavirenz/tenofovir/emtricitabine. After one month of treatment, prednisone was tapered with concomitant initiation of azathioprine (150mg daily). Her LAEs improved over the next year (Figure 1). Elevated IgG fraction declined. Her CD4 count has remained stable, and she is in excellent clinical condition.

## DISCUSSION

We present the first biopsy-confirmed case of a patient with HIV infection and overlapping AIH and PBC in the setting of antiretroviral therapy and immune reconstitution.

AIH has been rarely described in HIV-infected adults; a literature review identified thirteen previously reported cases [2–9]. Reports of immune reconstitution unmasking autoimmune liver disease in the HIV-infected patient have been described in only four patients previously [7, 9]. The challenge of distinguishing immune reconstitution from other etiologies of liver disease, such as drug toxicities and infections, commonly leads to interruption in ART, potentially placing patients at increased risk of HIV-related complications, especially in the setting of additional immunosuppressive therapy. In our patient, fluctuating levels of LAEs without medication changes suggested a non-pharmacologic cause, and liver biopsy with histologic confirmation allowed for confident continuation of ART.

In conclusion, immune reconstitution after ART initiation may reveal underlying infectious and non-infectious disease processes, including autoimmune hepatitis, and elevated LAEs in this setting should not immediately be attributed to medications. In cases of persistent, mildly abnormal liver enzymes, early liver biopsy is important in differentiating causes and should be pursued when other less-invasive tests are inconclusive. Histological confirmation can prevent ART interruption and its related risks. Standard treatment of AIH/PBC appears to be effective and safe in HIV-infected adults.
